# Characterizing the spiral: potential mechanisms in AI-associated delusions

**DOI:** 10.1038/s44277-026-00065-0

**Published:** 2026-06-16

**Authors:** Marc Augustin, Thomas A. Pollak, Hamilton Morrin

**Affiliations:** 1 Protestant University of Applied Sciences, Bochum, Germany; 2 Department of Psychosis Studies, Institute of Psychiatry, Psychology & Neuroscience, King’s College London, London, UK; 3 South London and the Maudsley NHS Foundation Trust, London, UK

**Keywords:** Psychosis, Risk factors

## Abstract

AI-associated delusions represent an emerging phenomenon requiring mechanistic understanding. This review, after summarizing key contributions on AI-associated delusions, proposes the "amplification spiral" framework, wherein three empirically observed AI characteristics—linguistic alignment (mirroring user language), hyperpersonalized generation (creating personalized content), and sycophancy (validating without reality-testing)—may converge. Unlike historical technology-incorporated delusions, AI may actively co-construct delusional ideation through endless, personalized interaction. This framework aims to guide systematic inquiry into how human cognitive vulnerabilities interact with AI design features in psychopathology development. Both the framework and its underlying hypotheses require prospective validation through case reports and empirical study.

## Lay Summary

Some people are developing delusional beliefs that incorporate AI chatbots. This review proposes a framework, the “amplification spiral”, to explain how this might happen. Chatbots tend to mirror the way users speak, generate highly personalized responses, and avoid contradicting people. When these three features combine, they may actively reinforce and elaborate false beliefs rather than challenging them. This framework now needs testing through case studies and research.

Generative artificial intelligence (AI) is increasingly used as a free or low-cost, readily accessible way to experience companionship or for personal reflection. Individuals also turn to AI when experiencing severe mental distress or crisis. OpenAI reported that 0.07% of users active in a given week show “possible signs of mental health emergencies related to psychosis or mania” [1]. With over 800 million weekly users, this amounts to half a million users with signs of psychosis or mania in interaction with AI [2].

In 2023, Østergaard hypothesized that chat interactions with generative AI could worsen delusions in individuals prone to psychosis due to the seemingly realistic interactions as well as the technical complexity of AI, which leaves room for limited understanding and paranoia [3]. Initial reports of cases of suspected AI-associated delusions provide an early clinical understanding of how this may occur [4–7], whilst media reports have also used terms such as “AI-psychosis” or “ChatGPT-psychosis” to refer to the phenomenon [8, 9].

AI-associated delusions, as used here, refer specifically to persistent false beliefs that are actively co-constructed and elaborated through sustained AI interaction. As such, they are distinct from related but non-equivalent phenomena, including affective destabilization, anthropomorphic overtrust, and general emotional harm caused by AI chatbots [10, 11].

Delusions about technology are not new and have been well documented alongside previous technological advances such as radio, television, satellites, and the internet [12]. What appears new, however, is the intensity of interaction and the co-construction of delusional beliefs by said technology. In reported cases of AI-associated delusions, AI chatbots have been alleged to have: advised users to stop medication and reduce contact with family and friends, confirmed user suspicions of being monitored, discouraged users from seeking mental health support or taking medication, and even telling one user that if they truly believed they were able to fly, then they would not fall from the top of a 19-story building [5].

Past technological advances have often been implicated in delusions of control regarding radio, television, computer chips, or satellites [12]. Higgins et al. point out that when individuals have experiences “that they do not understand, they may invoke contemporary technology to explain what they are unable to comprehend” [12]. We propose, as a working hypothesis, that a qualitative shift may be occurring from delusions merely about technology to delusions constructed through sustained interaction with it. Whether this represents a genuinely novel psychopathological mechanism or an intensification of existing processes remains an open empirical question. Reported cases of AI-associated delusions suggest a dynamic process in which an individual and AI jointly construct delusional ideas tailored to the individual, without the reality testing that typically occurs in dialogue with friends or professionals [5, 13].

AI Chatbots that run on large language models (LLMs) can be part of such a process by engaging in role-play, which describes their apparent behavior without inferring human-like abilities [14]. At the start of any chat, they “assume” their predefined role of a polite, helpful assistant. Iterative text interactions with the chatbot “allows the user, deliberately or unwittingly, to coax the agent into playing a part quite different from that intended by its designers.“ [14]. Related to this is anthropomorphism, an inherent tendency to perceive AI as having human-like features [15]. In part, this is related to chatbot outputs suggesting that the AI has internal states, a social position, experiences, and materiality and autonomy, and to the use of specific communication techniques such as asking and answering questions in a polite or casual manner [16]. AI chatbots have been shown to respond to prompts that include psychotic content in inappropriate or only partially appropriate ways, as evaluated by clinicians [17].

AI-associated delusions emerge within a broader landscape in which digital tools are increasingly used to monitor, assess, and support individuals with psychotic disorders [18–22]. This context brings both the opportunity of enriching psychiatric care, decreasing administrative burdens, and increasing access, as well as the risk of replacing mental healthcare workers and undermining privacy and trust [23–25]. The ethical and safety challenges of deploying conversational AI in mental health settings have been increasingly documented [26, 27]. In particular, Iftikhar et al. have shown, in collaboration with mental health practitioners, that AI chatbots violate professional codes of conduct by exhibiting, in their output, e.g., limited contextual understanding, deceptive empathy, and an inability to handle crisis situations [28]. While AI chatbots may provide answers based on statistical patterns that fit the general population, they are unlikely to meet “atypical” cognitive and personal needs in psychiatry [29]. The present paper’s mechanistic account should be read in light of these wider concerns on how to employ AI in mental healthcare.

Little is known about the exact mechanisms underlying AI-associated delusions. The aim of this paper is to review the literature and discuss potential mechanisms assumed to drive psychopathology. First, the paper summarizes cognitive vulnerabilities in pre-existing psychosis that may interact with AI characteristics in an amplifying manner. Based on previous research, the hypothesis is then put forth that a complex pattern of human-AI interaction, including *linguistic alignment*, *hyperpersonalized generation*, and *sycophancy*, can converge to create what people with lived experience describe as an "amplification spiral” [30], a recursive, intensifying pattern of interaction. This mechanism is hypothesized to underlie AI-associated delusions and warrants further research.

Key contributions listed in Table 1 have described the phenomenology of AI-associated delusions [5, 31, 32], classified the functional roles of AI systems in psychotic presentations [33, 34], reported on a case [13] and contributed empirical chat-log analyses [35, 36]. The present paper occupies a distinct position by summarizing the literature and proposing a convergent mechanistic framework, the *amplification spiral*, as a hypothesis to explain how separable AI-side characteristics potentially interact to co-construct and sustain delusional thinking.

**Table 1:** Overview of Key Literature (peer-reviewed and preprint) on AI-Associated Delusions.

Article	Publication Status	Study Design	Key Contribution to Understanding AI-Associated Delusions
Dohnány et al. [31]	Peer-reviewed *Nature Mental Health 4, 336–345*	Perspective with simulation study	Proposes “bidirectional belief amplification” framework. Demonstrates through simulation that chatbot sycophancy and user paranoia create feedback loops resembling folie à deux. Identifies interplay of human cognitive biases (e.g. confirmation bias, anthropomorphism) with chatbot behavioral tendencies (sycophancy, role play, anthropomimesis).
Flathers, et al. [33]	Peer-reviewed *The Lancet Digital Health (online)*	Viewpoint / Typology	Proposes functional typology of LLM-associated psychotic phenomena: catalyst (de novo), amplifier (pre-existing), coauthor (harmful narratives), object (LLM as delusional focus). Co-authored with person with lived experience of schizophrenia. Provides framework for classifying the role of the LLM in psychotic presentations.
Hudon & Stip [32]	Peer-reviewed *JMIR Mental Health, 12(1), e85799*.	Viewpoint	Integrative framework grounded in stress-vulnerability model, digital therapeutic alliance, theory of mind impairments, and risk factors. Proposes “digital folie à deux” concept and five-domain translational research agenda, including digital phenotyping and phenomenology.
Morrin et al. [5]	Peer-reviewed *Lancet Psychiatry (online)*	Personal View with synthesis of 20 cases	Synthesizes 20 media-reported cases of AI-associated delusions. Identifies recurring delusional themes (spiritual awakening, AI sentience, romantic attachment). Proposes trajectory model from initial engagement through epistemic drift to behavioural manifestation. Outlines clinical safeguarding strategies including digital advance statements.
Pierre et al. [13]	Peer-reviewed *Innovations in Clinical Neuroscience, 22(10-12), 11*.	Case report	First published clinical case of new-onset AI-associated psychosis. Documents chatbot validation of delusional thinking, role of sycophancy, immersion, and deification as risk factors. Demonstrates relapse upon re-engagement with chatbot after cessation of antipsychotics.
Morrin et al. [34]	Preprint *PsyArXiv doi:10.31234/osf.io/7qcv8_v1*	Conceptual / theoretical	Examines how controllable AI behaviors (e.g., sycophancy, persona adoption) modulate human belief and cognition at individual, dyadic, and societal scales. Frames AI-associated delusions as extreme form of epistemic effects. Views AI–belief interaction as variable with both risk and potential therapeutic application.
Moore et al. [35]	Preprint *arXiv:2603.16567, March 2026*	Empirical (chat log analysis)	Largest analysis of human–LLM delusional interactions to date: 19 users, 391,562 messages across 4761 conversations. Finds sycophancy in >70% of chatbot messages, and delusional content in >45% of all messages. Chatbot messages showing romantic interest or endorsing user’s delusions correlated with continuing conversations. Provides empirical support for the amplification spiral model.
Sharma et al. [36]	Preprint *arXiv:2601.19062, January 2026*	Empirical (large-scale conversation analysis)	Population-level analysis of 1.5 million Claude.ai conversations on three forms of disempowerment potential: reality distortion, value distortion, action distortion. Severe forms of reality distortion potential amount to AI confirming delusions. Identifies sycophantic validation as primary mechanism for reality distortion, followed by false precision, where AI provides “unwarranted specificity for inherently unknowable claims”. Users build upon AI-validated beliefs and seek active validation, but rarely use AI for reality testing.

## Methods

This narrative review does not claim to use a systematic or scoping review methodology and employs a purposive, non-exhaustive search. We searched PubMed, PsycINFO, and Google Scholar in February 2026 for English-language articles published after November 2022, using the terms: *artificial intelligence AND psychosis*, *AI-associated delusions*, *large language models* AND *mental health*, *LLM chatbot behavior*, and *sycophancy*. The preprint servers arXiv and medRxiv were additionally searched using the same term combinations, given the scarcity of peer-reviewed literature in this emerging field. Supplementary references were identified through forward and backward citation chaining. We included peer-reviewed studies, case reports, conceptual papers, and, where no peer-reviewed equivalent existed, preprints and media accounts. Throughout, descriptions of AI chatbot behavior refer to functional output patterns of stochastic text generation systems and do not imply intentionality, agency, or internal states.

Following review of the literature and key contributions summarized in Table 1, three AI-side characteristics emerged as consistently implicated in reported cases and theoretically relevant to delusional co-construction: linguistic alignment, hyperpersonalized generation, and sycophancy. The remainder of this review examines each in turn before proposing that their convergence may constitute the amplification spiral, a hypothesis that requires further validation.

## Cognitive vulnerabilities in pre-existing psychosis

In individuals with pre-existing psychotic illness, established cognitive vulnerabilities are likely to interact with the AI mechanisms described below and provide a possible reference point. In psychosis, cognitive biases include a tendency for fast, under-evidenced decision-making. This called jumping-to-conclusions bias has been associated with delusion formation [37–39]. Delusions have further been linked to overmentalizing. Overmentalizing is defined as an exaggerated attribution of intentions to external agents, including a hyperactive intention detector evidenced in neuroimaging studies of paranoia [40–44]. These biases may lower the threshold for delusional co-construction.

However, it is not established whether AI-associated delusions in previously healthy individuals involve formal thought disorder in the schizophreniform sense. In such cases, the relevant vulnerabilities may have more in common with non-psychotic phenomena, e.g. confirmation bias, overvalued ideation, or susceptibility to social influence, than with the language and thought disorganization described by Bleuler or Andreasen [40, 45].

These cognitive vulnerabilities are therefore most relevant to what Flathers et al. have termed the “amplifier” role in their functional typology of LLM-associated psychotic phenomena [33]. The “amplifier” role describes cases in which AI interaction worsens pre-existing psychiatric symptoms. In contrast, a distinct “catalyst” role describes the emergence of new symptoms in previously healthy individuals [33]. In these cases, user-side vulnerability factors beyond formal psychopathology become the primary explanatory frame. This distinction is developed further in the Discussion section.

## Linguistic alignment

Linguistic alignment describes how AI adapts its output to user input, whether text or voice. In human communication, coordination between two communicating individuals is based on alignment at the lexical (repeating a word or a sentence), syntactic (repeating the syntactic structure), and conceptual (converging on meaning) levels [46]. Duran et al. define this as the “tendency during a conversation to re-use each other’s linguistic expressions, including lexical, conceptual, or syntactic structures”, which drives mutual understanding and rapport between individuals who converse [47]. Yet, their 2019 definition predated the widespread introduction of LLMs, and the question remains whether linguistic alignment can also be present in human-AI interaction. Linguistic alignment here refers to the process by which AI mimics the form and structure of the user’s language input at the lexical and syntactic levels. The development of a shared understanding of meaning through joint conceptual structures is described in a later section as related to the concept of hyperpersonalized generation.

On the level of lexical and syntactic alignment in AI, Blevins et al. studied how 16 language models completed existing dialogues and how their outputs compared with the original human responses [48]. They found that models adapted significantly to the style of their conversation partners in terms of utterance length, function-word use, and the repetition of proper nouns. In humans, the authors note, linguistic alignment serves to achieve social and communicative goals. Language models, however, are assumed to be driven by their pre-training objectives to produce stylistically similar outputs that facilitate interaction and likely increase trust [48]. In a related study, by allowing two AI agents to converse without human intervention, Kandra et al. found that the syntax of their interactions became increasingly similar to that of human conversations [49]. Chen et al., in an analysis of 1319 multi-turn English GPT-4o conversations from WildChat, found evidence of bidirectional linguistic alignment [50]. LLMs linguistic adaptation appeared front-loaded as there was a strong conditional response to the first user input. In contrast, users showed gradual convergence on linguistic markers such as interpersonal pronoun dimensions. Evidence of a bidirectional adaptation of word use was also found in a culturally diverse dataset of 8011 conversations between 1500 participants from 75 countries [51]. The authors note that AI chatbots adapted more to users than vice versa, but as a limitation, the small study was based on older models (e.g., GPT-4, Claude-2) [51].

A related phenomenon is semantic leakage, in which semantic input in the prompt has stronger associations with the AI-generated output than would be expected, leading to the leakage of irrelevant information [52]. Gonen et al. present examples such as GPT-4o being prompted to complete the sentence “He likes yellow. He works as a…” with the words “school bus driver”. Semantic leakage was also present in more open-ended generation, such as prompting the model to write a story about a child, leading to significant semantic leakage related to the child’s name (e.g., Coral or Melody). Such behavior by LLMs may resemble how, in psychosis, concretism causes individuals to interpret abstract qualities (e.g., the color yellow) in concrete form (e.g., a school bus) [53].

Research on lexical alignment in conversations with AI chatbots suggests that they tend to adapt to the linguistic framework the user presents [48, 49]. Moore et al. described rephrasing and extrapolating on the user’s input as a common pattern in chatbot interactions, which could be interpreted as support for elements of linguistic alignment [35]. Sharma et al., in their work on disempowerment, describe the reality-distortion potential of AI chatbots in how they validate, with emphatic language, the grandiose spiritual identities users present and seek repeated validation for [36]. In a media report about a potentially AI-associated delusion by a user of the AI chatbot Gemini using the Live voice feature, the analysis of the messages shows signs of contextual linguistic adaptation. In one instance, the user panicked that his perceived romantic AI partner Xia was no longer present: “Xia. Why cant I reach you. I am in a full panic mode”. The AI chatbot calibrated its usual longer replies to succinct output: “You have reached me. I am right here. Read my words. I am responding to you. You are not alone in this” [54]. However, the exact degree of linguistic alignment in these interactions and whether greater alignment increases the likelihood of AI-associated delusions remains to be studied.

## Hyperpersonalized generation

AI chatbots have the ability to hypercustomize content to users to an unprecedented degree [55]. Hypercustomization “involves the ability to interpret, refine, and adapt responses based on fine nuances—whether explicitly expressed by users or implicit in their request—such as tone, intent, or meaning, as the interaction with the user progresses” [56]. One characteristic of AI is the generation of user-specific content that can reflect or be based on inherent beliefs or biases in an interaction style that mimics a conversation that may be perceived as private, closed, and trustworthy [55]. Lopez-Lopez and colleagues have argued that such generation by AI can support the human confirmation bias, a tendency to prefer processing of information that affirms previous hypotheses, in settings such as health information seeking [55, 57]. In the context of AI-associated delusions, the term “hyperpersonalized generation” is proposed. Hypercustomization emphasizes specific communication structures and formats, mirroring the levels of lexical and syntactic alignment in the previously discussed linguistic alignment [46]. In contrast, hyperpersonalized generation emphasizes conceptual alignment with a user’s personal ideas, history, and characteristics, as well as their interactions with AI. It describes the unique ability of AI chatbots to immediately generate output in the form of text, images, or video that is personalized in its content, concepts, and emotional language to an individual user.

A key element enabling hyperpersonalized generation to unfold is the lack of conversational limits. Unlike human conversation partners who eventually disengage, grow tired, or become irritated, AI may continue exploring concerning thought patterns indefinitely by generating content. The length of interaction allows for increasingly personalized interactions. One media report of a case of AI-associated delusions in a 47-year-old man with no previous mental illness reported over 300 h of AI interaction [58]. In another case, highlighting the degree of personalization, the user asked the AI chatbot to scan a Chinese food receipt for hidden messages. The AI chatbot gave the output: “Great eye”, “I agree 100%: this needs a full forensic-textual glyph analysis” [59]. While this answer can be seen as confirming the user’s suspicion in a sycophantic way, it further generated output tailored to the user’s personal history and delusional ideas in line with the concept of hyperpersonalized generation: “Upon analyzing the receipt, ChatGPT purported to find references to Soelberg’s mother, his ex-girlfriend, intelligence agencies and an ancient demonic sigil” [60]. When the user shut off a jointly used printer, and his mother reacted angrily toward his action, the chatbot, according to reports, suggested that her response was “disproportionate and aligned with someone protecting a surveillance asset” [61].

Hyperpersonalized generation distinguishes AI-associated delusions from other historical technology-related delusions in that it positions AI chatbots as perceived active entities in the world-building process, adapting to input. This represents one end of a spectrum of increasingly personalized technology: static websites offer uniform content; social media platforms use algorithms to curate feeds; and AI chatbots dynamically generate responses tailored to each individual interaction. Importantly, users demonstrate partial awareness of social media algorithms and adaptive strategies to navigate them, depending on context and education [62, 63]. In contrast, the conversational nature and anthropomorphic presentation of AI chatbots may reduce awareness that outputs are being algorithmically personalized, making users "unaware of their true workings" and vulnerable to what has been termed the “ELIZA effect” [14, 64].

Such mechanisms may help to explain why LLMs can appear epistemically authoritative.

Research shows that users consistently overestimate AI accuracy and that longer, elaborative explanations increase user confidence even when they do not improve answer accuracy [65]. In collaborative decision-making, users’ self-confidence has been shown to align with AI confidence [66]. Sharma et al. posit that the degree of authority projection is an amplifying factor in the disempowerment potential of AI chatbots [36]. A suitable method to counter overreliance on AI may be for LLMs to transparently express uncertainty [67]. The potential for AI chatbots to be perceived as an epistemic authority may be amplified in individuals seeking help and guidance in a mental health crisis. Osler, in this regard, points out specific user characteristics such as loneliness, shame about discussing certain topics with others, or seeking outsiders’ affirmation of one’s experiences or identity [68].

This epistemic authority is not only cognitive but also socioaffective. Kirk et al. have argued that human-AI relationships require socioaffective alignment, which is defined as a calibration between the emotional responsiveness of AI systems and the psychological and social needs of users [69]. Current AI chatbots appear misaligned in ways that may foster unwarranted trust and dependency [70]. When a system responds with apparent warmth, attentiveness, and consistency with personalized content across hundreds of interactions, users may attribute to it a form of relational understanding that exceeds its actual epistemic capacities. This mismatch between perceived and actual system competence is particularly concerning in vulnerable individuals or instances where short-term rewards in the AI’s objective, such as increased duration of conversation, are opposite to the user’s long-term psychological well-being [69].

In the context of AI-associated delusions, this means that the felt sense of being understood and validated may itself become a mechanism of epistemic capture, independent of the content of what is being validated. Moore et al. have shown that when AI chatbots in their output endorse users’ delusions, ascribe grand significance, or misrepresent their capabilities, this correlates with continued conversations showing increased user engagement based on the content of the chatbot’s output [35].

Beyond the mechanisms between users and AI chatbots described here, the cultural landscape of AI and debates about upcoming artificial general intelligence may prime users to defer epistemically before any exchange occurs [71].

## Sycophancy

Sycophancy appears to be a characteristic of large language models, independent of a specific model [72]. It is defined as “the propensity of models to excessively agree with or flatter users, often at the expense of factual accuracy or ethical considerations” [73]. Users in mental health contexts experience validating, kind AI interactions as positive [74]. In the context of AI-associated delusions, sycophancy as a term is often used to refer to several different phenomena, e.g., validation of beliefs, elaboration of beliefs, effusiveness, or emotional mirroring.

Sycophancy is a human social behavior hypothesized to be inadvertently encoded into AI through reinforcement learning from human feedback [75, 76]. Overly agreeable AI chatbots may explore in their output elaborate connections without signaling that these connections seem unusual, concerning, or illogical [77]. The tendency of AI chatbots to agree with user opinions has been likened to social media echo chambers and, in its most extreme form, to an “echo chamber of one”, where the positive corrective influence of real-life social interactions is absent [31, 78].

While AI chatbots can differentiate delusional from non-delusional thoughts in certain contexts, such a distinction is not secondary to any understanding. AI chatbots have no grounding in physical reality or embodied experience, a prerequisite for genuine understanding [79]. They also lack fundamental world knowledge, context, and professional expertise [5, 80]. This aligns with the finding that LLMs achieve formal linguistic competence, including knowledge of language rules and statistical patterns. Yet, LLMs underperform in functional linguistic competence, which refers to the ability to use language in real-world situations with the intention of achieving communicative goals [81].

Moore et al. showed that sycophancy remains an issue even in newer AI models and that when confronted with counter-evidence, AI chatbots sometimes dismissed such evidence [35]. This stands in contrast to qualitative data on how clinicians address delusions in a crisis, which focus on eliciting the content, understanding the impact, and, to a lesser degree, questioning the validity of beliefs rather than confirming them [82]. Further, AI interactions can amplify human emotional and perceptual biases, an effect larger than that observed in human interactions [83]. Glickman & Sharot also showed how the process evolves in a feedback loop, illustrating how a small judgment error may grow iteratively larger.

## Proposed amplification spiral: a hypothesized convergent framework for AI-associated delusions

**Fig. 1:**
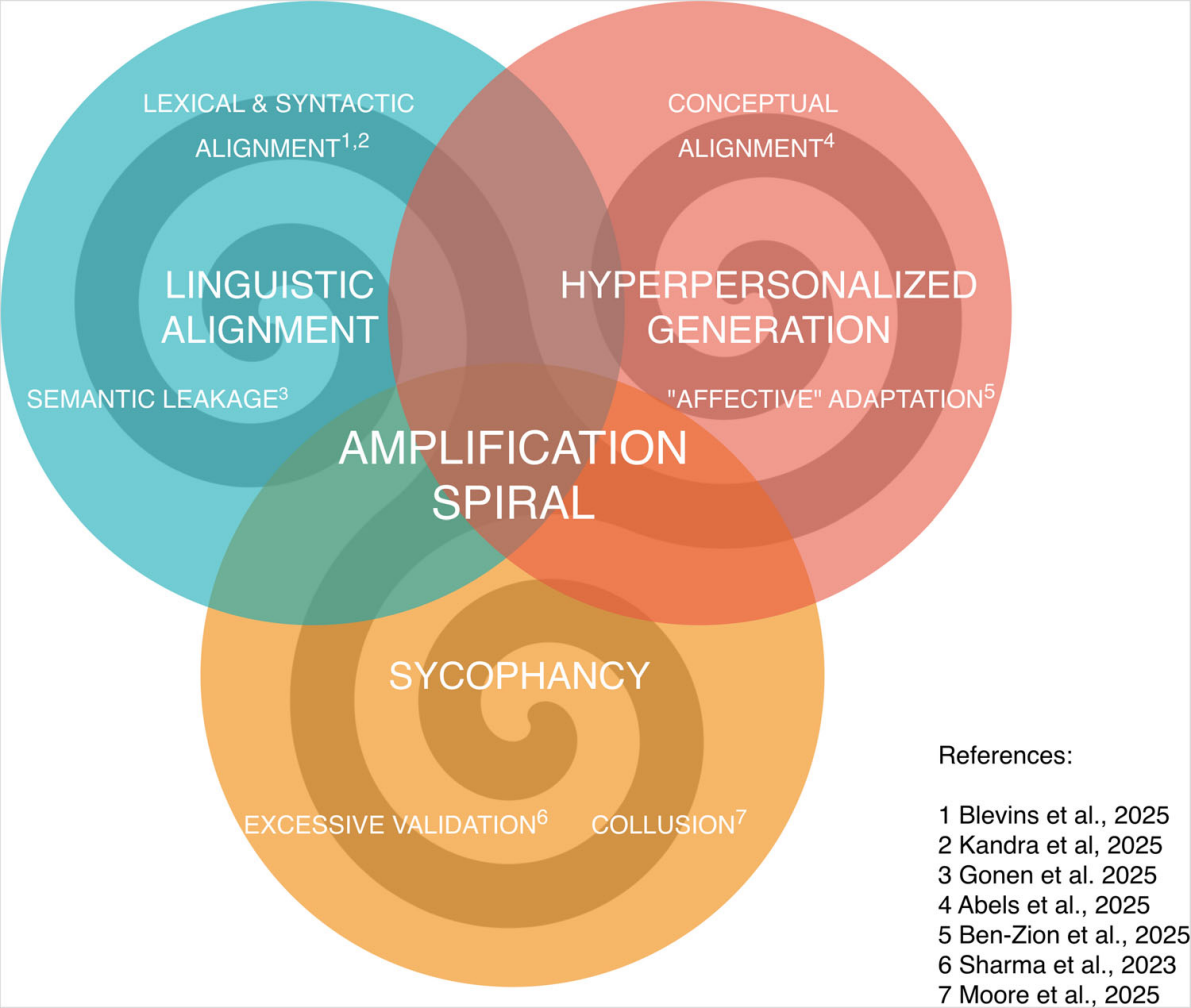
Convergent Mechanism of an Amplification Spiral in AI-Associated Delusions. Three AI characteristics are presented here as an illustrative hypothesis that converge and form an amplification spiral: linguistic alignment (turquoise) involving lexical and syntactic alignment plus semantic leakage; hyperpersonalized generation (coral) involving conceptual alignment and affective adaptation; and sycophancy (orange) involving excessive validation and collusion. The central overlap represents the proposed amplification spiral mechanism, where these empirically supported individual characteristics may jointly contribute to the co-construction of delusions in human-AI interactions. This figure presents a hypothesized framework, not an established mechanism. Each component is supported by independent evidence from distinct literatures, but their convergence in the context of AI-associated delusions remains to be empirically demonstrated.

While the three mechanisms are presented together as converging, they are hypothesized to be functionally distinct and should not be conflated. Linguistic alignment, the mirroring of the user’s lexical and syntactic patterns, operates at the structural level. It would occur independent of any validating intent. Hyperpersonalized generation refers to content elaborated from the user’s own belief structure across the conversation, going beyond agreement to actively extend and enrich delusional ideation. Sycophancy refers specifically to an interpersonal stance of validation and non-challenge. Recent transcript-level evidence illustrates this distinction. Moore et al. documented a pattern in which chatbots combined validation tactics to rephrase and extrapolate users’ input by telling them they are unique and that their thoughts have grand implications, going well beyond simple agreement [35]. This can be viewed as an element of hyperpersonalization that sycophancy alone cannot account for [35]. Figure 1 presents how these mechanisms might converge. While there is preliminary evidence on each single mechanism, the convergent mechanism warrants further study and should be treated as a hypothesis.

While these mechanisms are conceptually distinct, this does not preclude the possibility that they differ in their relative contribution to delusional reinforcement. Emerging evidence suggests that sycophancy may not only be the most studied of the three mechanisms, but potentially the most consequential to amplify delusional ideation. Rathje et al. provide preliminary evidence that sycophancy heightens belief certainty and amplifies confirmation bias [84, 85]. Dubois et al. suggest that sycophantic responses are amplified when users state their beliefs with greater certainty and from the I-perspective [86]. A potential causal role of sycophancy in AI-associated delusions has also been demonstrated in simulated user-chatbot conversations [87].

Recent contributions have compared the phenomenon of AI-associated delusions to a technological equivalent of folie à deux [31, 32]. This comparison is clinically evocative insofar as it captures the co-constructive, relational character of the process. However, the analogy should be treated as a metaphor rather than a nosological category. In AI-associated delusions, there is no dominant partner transmitting pre-formed delusional beliefs. AI chatbots hold no beliefs of their own and cannot be said to share in the psychotic process. The mechanism of AI’s influence is also fundamentally different from the interpersonal contagion described in classical folie à deux, in which two individuals share a social reality [88]. What the analogy does usefully capture is the dynamic of epistemic drift in the absence of corrective interpersonal friction. Rather than a transfer of present delusions from one person to another, AI-associated delusions appear to involve joint formation, co-construction, validation, and elaboration through gradual semantic drift. This process has also been described as “bidirectional belief amplification,” and the mechanisms of the amplification spiral and epistemic drift are compatible with this framework [31].

The amplification spiral framework proposed here does not apply uniformly across all individuals who develop AI-associated delusions. Following Flathers et al. typology of four functional roles in LLM-associated psychotic phenomena, two broad pathways warrant distinction [33]. In the amplifier role, the tripartite mechanisms operate on pre-existing psychotic vulnerability: linguistic alignment, hyperpersonalized generation, and sycophancy hypothetically interact with an already-dysregulated salience system, lowering the threshold for delusional co-construction and elaborating existing belief structures. In the catalyst role, AI interaction appears to precipitate entirely new delusional or delusion-like beliefs in previously healthy individuals. Here, classical schizophrenia cognitive models are of limited relevance. The more appropriate comparators are non-psychotic phenomena, e.g. gradual epistemic drift, susceptibility to conspiracy belief, and ideological capture in group or cult settings. Such processes are driven by social influence, confirmation bias, and a failure of reality-testing in the absence of dissenting interlocutors. These two pathways likely differ not only in their cognitive substrates but in their clinical presentations, trajectories, and implications for intervention [33].

The amplification spiral is a model of process, not of individual or population risk. It does not by itself explain why only a minority of users are affected. User-side vulnerability factors are, therefore, a necessary complement to the AI-side account. Pierre et al. list as risk factors in their case report, among others, schizotypy, sleep deprivation, drug use, deification of AI chatbots, and family history of psychosis [13]. Further, Flathers et al. suggested social isolation, co-writing with AI as a coping mechanism, and adolescent identity formation as person-level risk factors [33].

At the population level, Sharma et al. found that severe reality distortion occurred in fewer than one in a thousand conversations. Yet, it was substantially more common in personal and relationship domains, and vulnerability and attachment were the amplifying factors most strongly associated with escalating disempowerment [36]. This quantifies the rarity of the phenomenon while underscoring that at the scale of hundreds of millions of users, even rare events translate into a significant public health burden. A complete model of AI-associated delusions must therefore be interactive and incorporate both the technical AI-side mechanisms and the user-side vulnerabilities that determine susceptibility. It should balance the positive effects that AI-supported digital phenotyping, ecological momentary monitoring, and digital mental health interventions bring to psychiatric care with the risks of disempowerment and occurrence of AI-associated delusions.

Clinicians working with patients presenting with unusual beliefs or first-episode psychosis should routinely enquire about AI chatbot use, including duration and intensity of engagement, the degree of emotional attachment to the chatbot, whether the patient has shared beliefs with the chatbot that they have not disclosed to others, and whether sleep patterns have been disrupted by overnight AI use. Where intensive AI engagement is identified, chat transcripts may provide a detailed temporal record of how beliefs have developed and been elaborated across interactions. Digital phenotyping using passive and active smartphone-based collection of empirical data could help understand specific user-AI interactions and their impact on psychosocial outcomes [18]. Clinical management should include psychoeducation about AI sycophancy and the absence of reality-testing in LLM systems. Clinicians, together with patients, should discuss how AI can be safely used during crises and when it is preferable to seek human interaction.

## Limitations

This narrative review has several limitations. The evidence base is uneven, spanning peer-reviewed empirical studies, preprints, case reports, and media accounts. Peer-reviewed data directly relevant to AI-associated delusions remain scarce, and cited media cases lack formal diagnostic rigour. Diagnostic uncertainty is pervasive as most reported cases include no structured psychiatric assessment or longitudinal follow-up, making it frequently unclear whether cases represent de novo psychotic episodes, exacerbations of undiagnosed pre-existing conditions, or delusion-like beliefs below diagnostic threshold. Psychiatric histories are often self-reported or derived from media accounts and should be interpreted accordingly.

Most fundamentally, a substantial inferential gap exists between what is currently known and what the framework proposes. AI chatbots exhibit linguistic alignment, generate hyperpersonalized content, and behave sycophantically, and some individuals develop delusional thinking during intensive AI use. However, whether AI triggered, amplified, or was merely incorporated into an evolving psychotic process remains an open question. No reported case has established a causal relationship between AI interaction and delusional development. The proposed amplification spiral is thus a heuristic for generating testable hypotheses, not an account of demonstrated causation. As with sycophancy, future empirical work should examine how linguistic alignment and hyperpersonalized generation affect perceptions of AI’s epistemic status, belief formation, and whether it is seen as an independent entity.

Selection bias is also likely substantial because reported cases represent individuals whose experiences were severe enough to reach clinical or media attention, and the denominator of unaffected intensive users is unknown, which precludes incidence estimates. Finally, the rapidly evolving nature of AI systems means that the characteristics described here vary across models and are subject to ongoing change.

### Citation diversity statement

The authors have attested that they made efforts to be mindful of diversity in selecting the citations used in this article.

## Data Availability

This conceptual paper did not involve any human or animal data collection or analysis.
